# Neuroexergaming for MCI: Coin-play in the interactive Physical and Cognitive Exercise Study (iPACES)

**DOI:** 10.1093/geroni/igaf122.3816

**Published:** 2025-12-31

**Authors:** Winry Devenport, Valerie Needham, Paul Arciero, Cay Anderson-Hanley

**Affiliations:** Union College, Schenectady, New York, United States; iPACES LLC, Clifton Park, New York, United States; iPACES LLC, Clifton Park, New York, United States; Union College, Schenectady, New York, United States

## Abstract

Older adults face challenges to maintaining cognitive function and a paramount goal is stemming decline into Alzheimer’s Disease and related dementias (ADRDs). Physical exercise is a known protectant and may even slow changes (Kramer et al., 2018). Combining physical activity with mental exercise seems to further enhance benefits, as in a pedal-n-play neuro-exergame: iPACES (an interactive physical and cognitive system; Anderson-Hanley et al., 2018). Research continues to explore how to tailor such interventions to maximize benefits to specific brain functions. Herein, the impact of coin collection on task performance was examined. This gaming feature was added to increase interest in a structured mental exercise program for those with mild cognitive impairment (MCI). As part of a clinical trial, 80 households were enrolled in iPACES; both an MCI patient and a normative caregiver (CG) were followed for a year to examine the impact of pedal-n-play. Households were provided an under-table pedaler and a tablet to play iPACES multiple times per week. Over 3,000 rides were recorded and analyses revealed a significant interaction effect between MCI vs. CG, and coins on vs. off (p < .001). Those with MCI performed worse when collecting coins vs. not, while CGs performed better. Thus, coin collection may engage attention for normative persons, while interfering with performance for MCI. Future research could examine possible causality. The implications of this finding for the current refinement of iPACES and other prescriptive digital therapeutics (PDTx) suggests the importance of intentional game design and prescription according to cognitive abilities.

